# Effects of *ad libitum* consumed, low-fat, high-fiber plant-based diet supplemented with plant-based meal replacements on cardiovascular risk factors

**DOI:** 10.29219/fnr.v63.1560

**Published:** 2019-05-21

**Authors:** Boštjan Jakše, Barbara Jakše, Jernej Pajek, Maja Pajek

**Affiliations:** 1 University of Ljubljana, Biotechnical Faculty, 1100 Ljubljana, Slovenia; 2 Barbara Jakše s.p., 1230 Domžale, Slovenia; 3 University Medical Center Ljubljana, Zaloška 2, 1525 Ljubljana, Slovenia; 4 Faculty of Sport, University of Ljubljana, Gortanova 22, 1000 Ljubljana, Slovenia

**Keywords:** nutrition, atherosclerosis, obesity, cholesterol, fat, weight reduction

## Abstract

**Background:**

Sustainable nutritional strategies to reduce risk factors of cardiovascular diseases are highly needed. Inclusion of meal replacements may increase adherence to plant-based diets (PBDs).

**Objective:**

The aim of this study was to test the effects of a transition from a western-type diet to a new nutritional paradigm with a PBD from predominately unrefined whole food sources, eaten *ad libitum* and including nutrient-enriched plant-based meal replacements twice daily.

**Design:**

This was a single-arm, prospective interventional trial for 10 weeks in 36 participants with extension to 36 weeks in 18 participants. The main endpoint was serum low-density lipoprotein (LDL)-cholesterol measured at baseline, after 10 weeks (phase 1), and after 36 weeks (phase 2). Secondary endpoints included total, non-high-density lipoprotein (HDL) and HDL-cholesterol, fasting glucose, uric acid, and insulin-like growth factor-1 (IGF-1).

**Results:**

The mean reduction in LDL-cholesterol was 0.6 (95% confidence interval [CI], 0.3–0.8) mmol/L (−15%, *P* < 0.001) at the end of phase 1, with no further change by the end of phase 2. Similar reductions were noted for non-HDL-cholesterol and total cholesterol. HDL-cholesterol was reduced by 0.16 mmol/L (95% CI, 0.1–0.2). There was a borderline reduction in fasting glucose (5.2 to 5 mmol/L in phase 1, *P* = 0.08) and a small significant rise in serum uric acid levels of 15 (95% CI, 1–28) μmol/L, *P* < 0.05. Median baseline value for IGF-1 concentration was 156 μg/L. Participants with baseline IGF-1 below median had a significant increase in IGF-1 value from baseline 110 ± 31 to 132 ± 39 at the end of phase 1 (mean change of +22 μg/L, 95% CI, 11–33, *P* = 0.001). Participants with baseline IGF-1 above median had no significant change in IGF-1. Significant reductions in body weight, body fat, and visceral fat were observed.

**Conclusions:**

Supplemented, unrefined PBD eaten *ad libitum* was effective in improving total and LDL-cholesterol, non-HDL-cholesterol, and IGF-1 in low baseline IGF-1 subgroup.

This trial is registered as NCT02905448 at https://clinicaltrials.gov, registered 19.9.2016, https://clinicaltrials.gov/ct2/show/NCT02905448.

## Popular scientific summary

- We explored the benefits of whole food plant-based diet supplemented with plant-based meal replacements in a heterogenous group of free-living participants.
- The diet reduced serum cholesterol and body weight and then maintained improved values in the prolonged second phase of the study.
- A viable support system secured high compliance with the diet.
- This nutritional pattern is effective in maintaining long-term general health and is sustainable in fast-paced conditions of modern western life.

A modern western diet contains large amounts of refined sugar, added salt, cholesterol, and saturated fats on one side, and low amounts of whole grains, fruits, vegetables, legumes, and nuts on the other. As such it plays a critical role in rising rates of type 2 diabetes, arterial hypertension, hyperlipidemia, obesity, and coronary artery disease (1, 2). Nutrition is one of the foundations of cardiovascular risk reduction lifestyle changes (3). In recent years, adopting a plant-based diet (PBD) has become increasingly popular. PBDs are appropriate for all stages of the life cycle, including pregnancy, lactation, infancy, childhood, adolescence, older adulthood, and for athletes (4). PBD interventions are effective in lowering plasma cholesterol (5) and reducing body weight (6, 7). Evidence suggests that PBDs may reduce the risk of coronary heart disease events by an estimated 40%, and the risk of cerebral vascular disease events by 29% (8). A number of studies compared the nutritional profile of various diets, and concluded that the healthiest eating habits include a healthy version of PBDs because they potentially provide the best prevention against various chronic diseases and weight control (9–11). However, there is a general public perception that PBDs are hard to adhere to, and innovative ways to reach sustainable plant-based nutrition in a modern fast-paced everyday life are needed.

## Present investigation

### Background

Adopting a PBD is demanding, and many patients face significant problems and obstacles in achieving this goal. Although adherence to PBDs seems to be similar to most of the other therapeutic dietary approaches (12), the transition to a PBD may be more successful with an inclusion of patients in a lifestyle optimizing program providing a rationale for a diet change through lectures, daily meal plans, and social support, including motivational sessions. An additional strategy to increase compliance and long-term success in diet programs is to include meal replacements (MR) in the daily dietary plan. The success of weight reducing diets was significantly improved with the inclusion of MR in overweight/obese free-living subjects, likely due to a simple preparation and general convenience (13). The systematic review of six randomized, controlled, MR intervention studies in overweight adults of at least a 3-month duration showed this to be a safe and effective way to achieve short- and long-term sustainable weight loss (14).

We developed a new nutritional paradigm, which combines four approaches: (1) PBD, (2) predominately whole food sources – unrefined, (3) eaten *ad libitum* to increase sustainability, and (4) inclusion of nutrient-enriched plant-based MR. With such a diet it can be easier for a modern, fast-paced person to maintain a healthier long-term PBD pattern. Although we previously reported on the effects of this nutritional paradigm and extensive lifestyle modification support program on body composition indices (7), the general health impact is not well established yet. Here we present the effects of this dietary approach on cardiovascular risk factors assessed in short- and long-term program participants.

## Method

This study was designed as an open-label, prospective, single-arm interventional study in a nonresident heterogeneous sample of self-selected and free-living subjects. They participated in a diet-optimizing program centered on PBD that lasted from October 2016 to July 2017. The primary outcome of the study was a change in serum low-density lipoprotein (LDL)-cholesterol concentration. Secondary outcomes included changes in total cholesterol, non-high-density lipoprotein (HDL)-cholesterol, triglycerides, fasting glucose, uric acid, insulin-like growth factor-1 (IGF-1) serum concentration, and body fat mass. Complete blood cell count and serum creatinine were examined as safety parameters.

### Subjects

We included participants older than 18 years regardless of their current weight. We excluded pregnant or lactating women, patients with dietary restrictions due to medical causes, patients with active malignant diseases, people who were already on PBDs, and competitive or top level athletes. Subjects were invited to participate in the study after spontaneously attending the introductory lecture based on information gathered by personal informal contacts, referrals from previous program attendees, and based on results posted on social networks. Each participant signed an informed consent form for the inclusion in the study. The study was conducted in accordance with the Declaration of Helsinki, and the protocol was approved by the Slovenian Medical Ethics Committee (approval document no. 0120-435/2016-3).

### Study diet and protocol

The baseline evaluation included a questionnaire-based survey of dietary habits, physical activity, comorbid diseases, and personal goals associated with participation in a diet-optimizing program. A questionnaire developed by the investigators was used and assessed the dietary habits with items defining the current intake of main food groups, a typical daily meal plan, dietary supplements, and diet-related health issues. Physical activity was assessed with three items in the questionnaire, defining the number of exercise units per week, the type of exercise, and the time spent.

The survey revealed that at baseline, most participants ingested on average two to four meals, which were reflective of a typical Western-type diet: most meals were composed of some animal-source food (cow’s milk, yoghurt, cheese, cottage cheese, meat from various sources, eggs, and fish) and refined wheat flour-based food (bread, pasta, and pastry). Food preparation included the use of various vegetable oils and fats. Unrefined and whole plant nutrients were largely absent from most meals, and a minority of meals included portions of fruit and vegetables. This data indicates that our participants were in general without any significant personal preference toward PBDs before entering the study. No ideological or philosophical arguments toward vegetarian diet choices or against animal use were mentioned.

The dietary intervention was executed in free-living conditions with participants engaging in their regular daily work and social activities. In the first phase, participants entered the lifestyle support program, including a dietary plan and weekly lectures with motivational sessions and peer interaction. The initial 10-week phase was followed by the second study phase that lasted for 26 weeks. Here, monitoring was limited to intermittent checks of dietary diaries, monthly monitoring of body composition, and additional daily or weekly personal communication to help them persevere in the new lifestyle. Dietary diaries and meal photographs were communicated to researchers on fortnight basis, and body composition measurements were done once weekly. These checks were used to correct and adjust the deviations from the dietary plan and to help participants prepare the meals.

Hour-long weekly lectures (executed 10 times in phase 1) about the rationale and guidance on how to attain the PBD and healthy active lifestyle were given to all subjects. The participants were encouraged to engage in at least two weekly sessions of 45 min of moderate-intensity exercise, to give participants the possibility to supplement their new diet with some minimal level of physical activity as a paradigm for a healthier lifestyle change. The guided 45 min of moderate-intensity exercise were organized for those who opted for it. During phase 1, approximately 80% of all participants attended these organized exercise sessions, while in phase 2 the number was at approximately 50%, as the participants became more independent and performed the prescribed exercise activities by themselves. We used social media to support the participants in following the targeted diet by posting testimonials, recipes, giving answers to their dilemmas about nutrition, and by posting professional summaries and advice related to nutrition and chronic diseases.

The dietary plan included two plant-based MR (15 g of pea protein per portion) and three conventional meals based on starch nutrients (potatoes, sweet potatoes, rice, oatmeal, whole-grain pasta, beans, peas, lentils, and similar ones), fruits (seasonal fruits and various berries), and non-starchy vegetables (color and leafy vegetables). Spices, tomato sauce (without oil), and one regular-sized spoon of whole flaxseeds (but added as grinded) were recommended as well. No more than 5–6 g of iodinated sea salt per day was recommended. All milk and dairy products, vegetable oils, and fats were excluded from the diet. Meat was allowed (but not recommended) in a small portion once a week to relieve social pressures on participants, which they often encountered from their circle of influence (i.e. family, friends, and coworkers). In addition, two herbal beverages containing black, green, and hibiscus tea extracts daily were added to the intervention.

The total macronutrient composition of the intervention diet (phase 1) was approximated to 20% protein, 65% carbohydrates, and 15% fat. The dietary fiber content in phase 1 was approximately 40–45 g per day. Both MR and conventional meals were allowed to be consumed *ad libitum* – to full satiety. No calorie count or limits were instituted. Calorie estimation was based on the recommended dietary meal plan and dietary diaries and photographs, using ESHA Food Processor Nutrition Analysis Software (http://www.esha.com). Average energy intake was estimated to 1,600 kcal per day. The dietary program was self-financed, but researchers were able to control the compliance of the entire targeted meal plan. The exact composition of the intervention diet (phase 1) is given in Table 1.

**Table 1:** Composition of the intervention diet in the first 10 weeks (phase 1)

Meal	Dietary plan	Macronutrient composition^†^	Calorie intake (kcal)^‡^
Breakfast	Instant herbal beverage MR (serving size three table spoons in water or optional soy milk), ground flaxseeds (one table spoon), oat meal (*ad libitum*), dates (two pieces)	25% protein, 55% carbohydrate, 20% fat	350
Morning snack	3 dcl of smoothie (spinach, berries, or other seasonal local fruits) or two to three portions of seasonal fruits	10% protein, 80% carbohydrates, 10% fat	200
Lunch	Instant herbal beverage Centered around starches; four to five food groups (whole grains: brown rice, pasta, buckwheat, millet, corn; legumes: lentil, bean, pea; tubers and pumpkins: potato and sweet potato; brassica: broccoli, cauliflower, kale, cabbage; color and leafy vegetables: tomato, green salad)	15% protein, 80% carbohydrate, 5% fat	500
Afternoon snack	Sandwich (whole-grain bread, humus/nut butter, tomato, kale, or cabbage) or millet with mixed berries or seasonal local fruits (if not already for morning snack)	20% protein, 60% carbohydrate, 20% fat	250
Dinner	Mixed green salad: green leafy vegetables, boiled potato, tomato, few walnuts, or what was left from lunch and always MR (serving size three table spoons in water or optional soy milk)	25% protein, 60% carbohydrate, 15% fat	300

† Overall (on average) estimated macronutrient composition: 20% protein (80 g), 65% carbohydrate (260 g), 15% fat (26 g), and 1,600 kcal.

‡ Calorie estimation was based on the recommended dietary meal plan and dietary diaries and photographs, using ESHA Food Processor Nutrition Analysis Software (http://www.esha.com). The MR used was Herbalife Nutrition European Free From Vanilla^®^ nutritional powder. The herbal drink used was Herbalife Nutrition Instant Herbal Beverage with Tea Extracts original flavor.

After 10 weeks, the participants were invited to the phase 2 of the study where we added EPA and DHA omega-3 fatty acid supplements (three tablets of 189 mg or one tablet of 567 mg per day), vitamin B_12_–methylcobalamin (1,000 μg 2–3 times a week), and dietary fiber supplements (3 times a day 5 g of fiber in the form of powder reconstituted in water; Herbalife Nutrition Oat apple fiber). The total fiber intake was recommended to increase to 60–65 g per day. A larger selection of a moderate amount of high-fat and high-protein whole plant-based foods (e.g. sesame seeds, avocado, soybean tofu, different nuts, and nut butters) on the conventional side (breakfast and afternoon snack) were added to the meal plan to relieve dietary constrictions and to improve the diversity of the diet. The total macronutrient composition of the intervention diet in phase 2 remained the same and was approximated to 20% protein, 65% carbohydrates, 15% fat, but had on average a slightly higher calorie intake of 1,700 kcal per day.

### Assessment of study endpoints

Study endpoints were assessed at baseline, after 10 weeks (phase 1), and at 36 weeks, at the end of the study (end of phase 2). Body composition was assessed by an 8-electrode medically approved bioimpedance body composition monitor (Tanita 780 S MA; Tanita Corporation, Tokyo, Japan). Body composition indices included body weight, body mass index (BMI), body fat mass percentage relative to total body mass, visceral fat rating (in arbitrary units associated with abdominal visceral fat cross-sectional area, where each unit equals 10 cm^2^ of visceral fat), muscle mass, and total body water. Before each bioimpedance test, the participants did not eat or drink for at least 1 h, exercise for at least 24 h, and urinate for at least 30 min. For biochemical assays, 10–15 mL of blood was taken after an overnight fasted state. Lipid and other biochemical parameters were measured with standard laboratory tests in certified medical biochemical laboratories (Synlab International^®^, Germany). Beckman Coulter^®^ analyzer AU680 was used for biochemical analyses. For LDL–cholesterol, the intra- and interassay coefficient of variation ranges were 1.36–2.26% and 2.34–2.71%, respectively. Linearity was secured within a concentration range of 0.26–10.3 mmol/L. IDS-iSYS chemiluminiscence assay based on monoclonal anti-IGF-1 antibody was used for the IGF-1 serum concentration measurement. The reportable range of assay was 10–1,200 ng/mL with intra- and interassay coefficients of variation up to 2.9 and 7.2%, respectively.

### Statistical analysis

Results are presented as means ± SD for normally distributed and as medians (range) for non-normally distributed variables. Values of endpoints at the end of phases 1 and 2 were compared to baseline with a *t*-test for paired samples and Wilcoxon signed-rank tests, as appropriate. Chi-square test was used for categorical variables. As the inclusion criteria did not restrict the selection of participants to overweight participants, we preplanned the subanalysis of main endpoints and body composition indices in two divided subgroups above and below the baseline BMI of 25. No results of any participant were excluded, and no interposition of missing values was done. IBM SPSS statistics application was used for all analyses; *P* < 0.05 was taken as the limit of statistical significance.

## Results

We were able to include 36 participants in the study, 25 females (69.4%) and 11 males (30.6%). Their demographic properties are shown in Table 2. All 36 participants finished phase 1, while 18 participants entered and finished phase 2. There were no significant differences in age, BMI, and baseline LDL-cholesterol in participants that have successfully finished phase 2 compared with the rest of the sample.

**Table 2:** Baseline demographic properties of participants in the study

Parameter	Whole sample (*N* = 36)	Females (*N* = 25)	Males (*N* = 11)	*P*
Age (years)	38.3 ± 9.9	37.9 ± 9.7	39.5 ± 10.9	0.66
Weight (kg)	81.4 ± 22.7	75.6 ± 22.8	94.7 ± 16.9	0.02
Height (cm)	171 ± 8	167 ± 4	179 ± 7	<0.001
Maximal lifetime weight (kg)^†^	85.8 ± 24.1	78.5 ± 22.1	102.6 ± 20.6	0.004
Maximal lifetime BMI (kg/m^2^)	27.8 (19.7–47.8)	25.6 (19.7–47.8)	32.2 (21.3–40.7)	0.05
Current BMI (kg/m^2^)	26.5 (19.1–48.5)	25.5 (19.1–48.5)	31.2 (20.4–34.8)	0.07
Smoking (*n* (%))	3 (8.3%)	2 (8%)	1 (9.1%)	1
Married or living with a partner (*n* (%))	23 (63.9%)	15 (60%)	8 (72.7%)	0.71
University educational level (*n* (%))	22 (61.1%)	16 (64%)	6 (54.5%)	0.72
Frequent exercisers^‡^ (*n* (%))	9 (25%)	5 (20%)	4 (36.4%)	0.41

† The maximal reported weight that a participant reached at any time during his/her life.

‡ Habitual personal workout for more than 3 times per week. Data is shown as mean ± standard deviation, median (minimum-maximum) and frequency (percent).

**Fig. 1:**
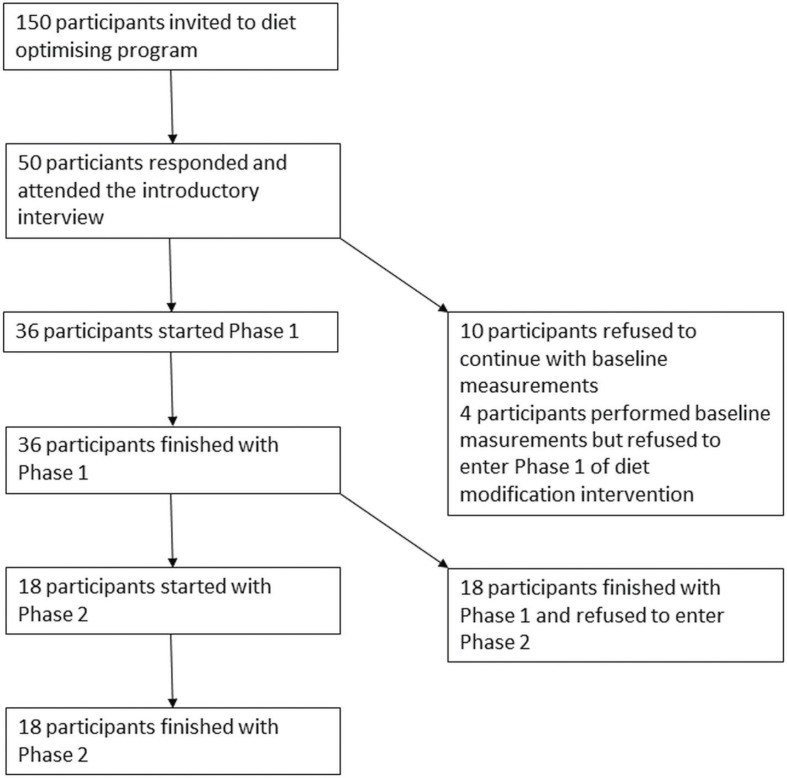
Flow of participants through study phases.

The flow of participants through study phases is shown in Fig. 1.

Changes in body composition were favorable (Table 3). Participants lost in average 2.6 kg in phase 1 and for those who participated in phase 2, an average weight loss of 3 kg was observed. This change was accompanied by significant reductions in body fat and visceral fat. Average BMI decreased to the normal range at the end of the study. There was a minimal significant change in muscle mass, which stabilized in phase 2.

**Table 3:** Body composition was measured at baseline, at the end of phase 1, and at the end of phase 2 with the bioimpedance method

Parameter	Whole sample (*N* = 36)		Subsample (*N* = 18)	
	Baseline	End phase 1	End phase 1	End phase 2
Weight (kg)	70.8 (51.4–132.1)	68.2 (48.2–119.1)***	66.4 (48.2–104.9)	63.4 (46.5–101.9)**
Body fat (%)	30.3 ± 10.1	26.3 ± 8***	26.5 ± 6.4	24.6 ± 5.5*
Visceral fat (arbitrary units)	5.5 (1–20)	4 (1–16)***	4 (1–16)	4 (1–15)*
BMI (kg/m^2^)	26.5 (19.1–48.5)	25.2 (17.9–42.2)***	24.7 (17.9–34.4)	23.4 (17.3–31.7)**
Muscle mass (kg)	48.8 (37.1–86.5)	48.7 (37.1–84.1)*	46.7 (37.1–77.9)	46.7 (38.6–75.3)
Total body water (L)	50.4 ± 6.4	53.1 ± 5.9***	52.7 ± 4.8	53.7 ± 4.1

* *P* < 0.05 compared with the previous study time point;

** *P* < 0.01 compared with the previous study time point;

*** *P* < 0.001 compared with the previous study time point. Data are shown as mean ± standard deviation for normally distributed variables and as median (minimum–maximum) for non-normally distributed ones.

**Fig. 2:**
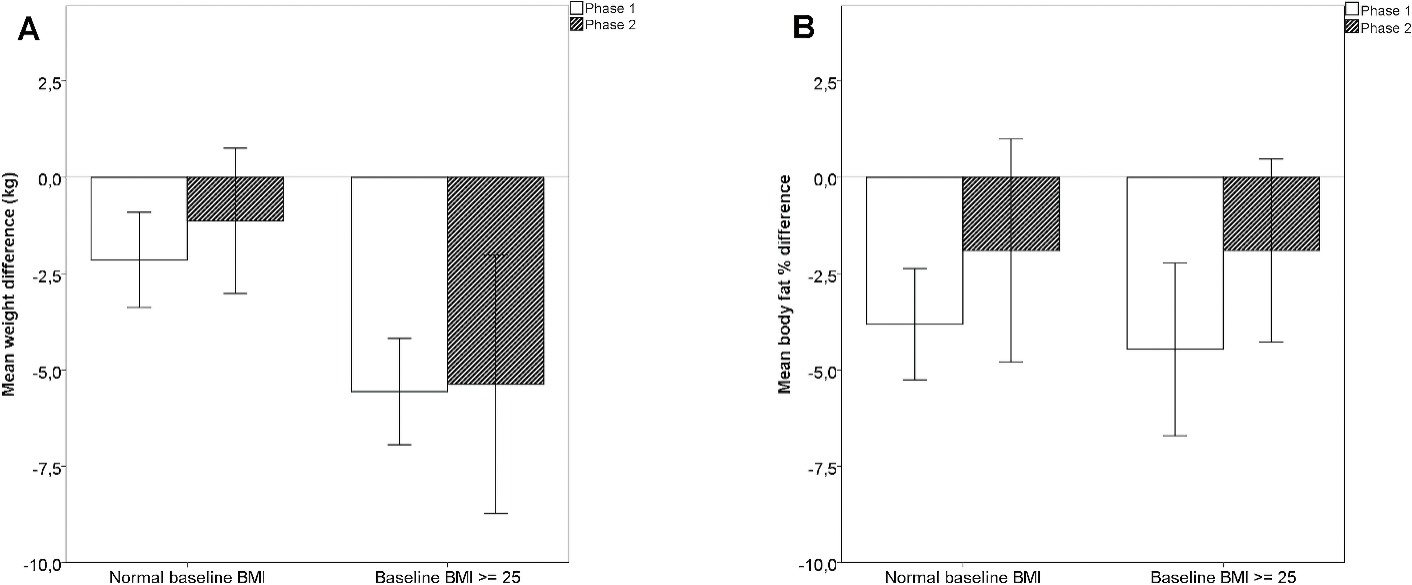
Changes in weight and body fat in body mass index subgroups. (A) Mean reductions of body weight according to baseline BMI. The differences between BMI subgroups are significant, *P* < 0.001 for phase 1 and *P* = 0.025 for phase 2. (B) Mean reductions in body fat percentage according to baseline BMI, the differences between BMI subgroups are not significant. Error bars represent 95% CI.

In participants with the BMI 25 or more mean reductions in body weight were significantly larger than in participants with normal BMI values, while mean reductions in body fat percentage did not differ significantly between these two BMI subgroups (see Fig. 2).

LDL-cholesterol as the main study endpoint was significantly reduced from 3.2 ± 1 to 2.6 ± 0.7 mmol/L in the first phase, and then remained stable in phase 2 (Table 4). The changes in other serum lipid parameters showed a significant reduction in total and non-HDL-cholesterol as well. The absolute reduction in LDL-cholesterol was much larger than the small reduction in HDL-cholesterol, which yielded a large reduction in non-HDL-cholesterol. Triglycerides did not show a significant change, while there was a tendency toward a small drop in fasting serum glucose level.

**Table 4:** Values of serum lipids, fasting glucose, and uric acid

Parameter	Whole sample (*N* = 36)			Subsample (*N* = 18)		
	Baseline	End phase 1	Mean difference (95% CI)	End phase 1	End phase 2	Mean difference (95% CI)
Cholesterol (mmol/L)	4.9 ± 1.2	4.1 ± 0.8***	0.7 (0.4–1)	4.2 ± 0.8	4.2 ± 0.8	–0.04 (−0.3 to 0.2)
LDL-cholesterol (mmol/L)	3.2 ± 1	2.6 ± 0.7***	0.6 (0.3–0.8)	2.6 ± 0.6	2.6 ± 0.7	0 (−0.2 to 0.2)
HDL-cholesterol (mmol/L)	1.3 ± 0.3	1.2 ± 0.3***	0.16 (0.1–0.2)	1.2 ± 0.3	1.2 ± 0.3	0 (−0.1 to 0.05)
Triglycerides (mmol/L)	1.3 ± 0.8	1.2 ± 0.6	0 (−0.2–0.2)	1.3 ± 0.7	1.3 ± 0.6	0 (−0.1 to 0.2)
Non-HDL-cholesterol (mmol/L)	3.5 ± 1.2	2.9 ± 0.8***	0.6 (0.3–0.9)	3 ± 0.7	3 ± 0.7	0 (−0.3 to 0.3)
Fasting glucose (mmol/L)	5.2 (4.2–9.9)	5 (4.3–7.1)^†^	N.A.	5.1 (4.6–7.1)	5.1 (4.4–6.7)	NA
Uric acid (μmol/L)	318 ± 78	332 ± 79*	15 (1–28)	330 ± 77	321 ± 86	−9 (−33 to 15)

CI, confidence interval for the difference between study time points;

* *P* < 0.05 compared with the previous study time point;

*** *P* < 0.001 compared with the previous study time point; NA, 95% CI for differences were not calculated due to a non-normal distribution of data.

† Reduction of fasting glucose in phase 1 did not reach statistical significance with a *P* of 0.08. Data are shown as mean ± standard deviation for normally distributed variables and as median (minimum–maximum) for non-normally distributed ones.

The mean reduction in LDL-cholesterol was 15 ± 17% and 13 ± 16% in total cholesterol in phase 1. In phase 2, the relative change in LDL-cholesterol was 1 ± 15% and in total cholesterol 2 ± 15%. In phase 1, the relative reduction of LDL-cholesterol was 18 ± 19% in participants with BMI < 25 (*N* = 13) and 14 ± 16% in participants with BMI 25 or more (*N* = 23, *P* for difference between the two BMI groups 0.53).

Baseline uric acid concentrations were significantly larger in men in comparison with women (387 ± 77 vs. 287 ± 57 μmol/L, respectively, *P* < 0.001). In phase 1, uric acid increased by 26 ± 61 (8 ± 18%) and 10 ± 27 μmol/L (5 ± 11%) in males and females, respectively, *P* = 0.42 for difference in the change between genders. In phase 2, uric acid changed for 1.5 ± 62 (1.3 ± 14%) and −14 ± 42 μmol/L (−4 ± 14%) in males and females, respectively, *P* = 0.54 for the difference between genders. In the subsample that finished both study phases, final uric acid did not differ significantly from the baseline value (321 μmol/L vs. baseline 309 μmol/L, *P* = 0.33).

The IGF-1 concentration measurement was available for 30 participants in the first study phase. At study entry, IGF-1 serum concentration was 156 ± 58 μg/L (range 60–262 μg/L) and at the end of phase 1 163 ± 49 μg/L (range 64–253 μg/L), *P* = 0.15. In the second phase, IGF-1 concentration measured in the subsample of 18 participants was 175 ± 40 at the beginning and 172 ± 37 μg/L at the end of the study (*P* = 0.64, *N* = 18). The median value for baseline IGF-1 concentration was 156 μg/L. Participants with a baseline IGF-1 below median had a significant increase in IGF-1 value from baseline 110 ± 31 to 132 ± 39 at the end of phase 1 (mean change of +22 μg/L, 95% confidence interval [CI], 11–33, *P* = 0.001). Participants with a baseline IGF-1 above median had no significant change in IGF-1 concentrations in phase 1 (baseline 201 ± 37, end phase 1 194 ± 38, mean change −8 μg/L, 95% CI, −21 to 6, *P* = 0.23). When a similar division of the sample, according to baseline IGF-1 values, was inspected in the second phase of the study, the participants with a baseline IGF-1 below median started and finished second phase with IGF-1 of 150 ± 31 and 156 ± 28 (*N* = 8, *P* = 0.56) and participants above median baseline IGF-1 started and finished second phase with IGF-1 of 195 ± 36 and 185 ± 39 μg/L, respectively (*N* = 10, *P* = 0.24).

There was no significant difference in serum creatinine at study entry (median 69, range 52–105) and at the end of phase 1 (median 67, range 57–95, *P* for comparison 0.17) and phase 2 (median 68, range 54–94 μmol/L, *P* for comparison 0.12). The blood leucocyte concentrations (in 10^9^/L units) were 6.4 ± 1.6, 6.1 ± 1.6 (*P* = 0.11) and 6.1 ± 1.3 (*P* = 0.73) at study entry, at the end of phase 1, and at the end of phase 2. The absolute blood lymphocyte concentrations were 2.2 ± 0.6 and 2.1 ± 0.5 (*P* = 0.02) and 2.1 ± 0.6 (*P* = 0.98) at study entry, at the end of phase 1, and at the end of phase 2. The mean difference in absolute lymphocyte concentrations at the end of phase 1 was −0.16 (95% CI, −0.02 to −0.3 × 10^9^/L), which was interpreted as a significant but clinically not important change. The hemoglobin concentrations (in g/L units) were 138 ± 11 and 139 ± 11 (*P* = 0.41) and 140 ± 11 (*P* = 0.32) at study entry, at the end of phase 1, and at the end of phase 2. We observed no adverse events associated with intervention in any phase of the study.

The adherence to participation in the program in phase 1 and in the reduced number of participants in phase 2 until the end of the study was 100%, as there were no dropouts from the program. This was a consequence of the enhanced and extensive support system as revealed in the methods section. Through regular communication and visits of participants we were able to closely monitor the dietary compliance. Although some individuals made occasional ‘mistakes’ (such as intake of excluded foods, occasional oil usage with salads, intake of bread with added ingredients of oil or milk), the vast majority followed the prescribed diet to the full.

## Discussion

Dietary changes are recommended as a first-line therapy for dyslipidemia and cardiovascular disease prevention in all individuals because they are safe and cost-effective (3). Our results show that a transition from a modern western-type diet to a low-fat, unrefined whole food PBD supplemented with plant-based MR, even if eaten *ad libitum*, enables significant and meaningful improvements in cardiovascular risk factors. Of special significance is our finding that the mean changes in LDL and non-HDL-cholesterol, which represent two most important lipoprotein risk factors, were both reduced for 0.6 mmol/L from the baseline values of 3.2 and 3.5 mmol/L. Already at baseline, this was close to the most recent guideline-suggested targets of 3 and 3.8 mmol/L for LDL and non-HDL-cholesterol, respectively (3). Additional benefits included a reduction of body fat percent and visceral fat, normalization of average BMI, and a trend of significant reduction in fasting glucose. Serum triglycerides showed a minimal non-significant reduction in the target range and there was a small increase in serum uric acid levels in phase 1. However, no significant elevations in uric acid were present at the study end in subsample of participants who finished both study phases.

Recent systematic review and meta-analysis of 30 observational studies and 19 clinical trials that examined associations between PBDs and plasma lipids showed that the consumption of vegetarian diets, particularly vegan diets, is associated with lower levels of plasma lipid, but not with decreased triglycerides compared with omnivorous diets in observational studies and clinical trials (5). In a nonrandomized study (without a control group) on 328 obese people who were not taking cholesterol-lowering medications, Fuhrman and Singer showed that in a 1-year plant-based intervention the average drop of LDL-cholesterol was 25% (from 4.4 to 3.3 mmol/L) and that triglycerides decreased by 40% (from 2.3 to 1.4 mmol/L), while the total cholesterol change for the interventional group was not reported (15). Our results extend this finding by showing a further meaningful reduction of LDL-cholesterol in participants with marginally elevated baseline cholesterol, even in the subsample of participants with normal BMI. This is a demonstration of the beneficial effect of predominantly unrefined *ad libitum* consumed PBD supplemented with MR, which, in our view, is a nutritional strategy very well suited to demands and time constraints of contemporary western lifestyle of the majority of working people.

A review of interventional studies using MR for weight loss as a part of a low calorie diet plan showed adherence of 50–70% with varied retention between 48 and 100% (16). Inclusion of MR may represent one of the solutions for improving long-term success of weight-reducing diets combined with standard lifestyle changes in overweight free-living subjects, probably due to a simple preparation and general convenience (13). As a nutrient-dense food, MRs help achieve and maintain nutrient adequacy without delivering excess calories (17). Our diet intervention included two plant-based MR in the daily meal plan and provides positive evidence for adding plant-based MR to PBD, extending the findings from abovementioned weight loss and calorie-restriction interventions in overweight subjects.

We included IGF-1 as an endpoint in this study due to associations of this hormone with cancer risk and metabolic syndrome (18, 19). Participants with a baseline IGF-1 below median significantly increased IGF-1 from baseline 107 to 129 μg/L at the end of phase 1 and participants with a baseline IGF-1 above median had a non-significant drop in IGF-1 concentrations. As there is a U-shaped relation of IGF-1 with all-cause mortality, these changes in both groups may be beneficial in the long term (20). In our study, median IGF-1 concentration of 154 μg/L was practically the same as the median value in a larger previous study, where baseline concentrations of IGF-1 below the median of 152 μg/L predicted future risks of impaired glucose tolerance and type 2 diabetes mellitus (21). From this viewpoint we regard the significant elevation of IGF-1 in a low baseline IGF-1 subgroup as a positive change, which is possibly associated with a reduced risk of developing the metabolic syndrome and with improved insulin sensitivity (19, 22).

Two main drawbacks of our study are the relatively low number of included participants and the absence of a control arm. In the absence of a control comparator group, we must consider other causes for the observed reductions of body weight, fat, and cholesterol and some changes in IGF-1 described above (23). Seasonal effects are not likely because recruitment and intervention were executed in all seasons of the year and we have not observed any seasonal differences. A statistical phenomenon of regression toward the mean is another possible cause of significant differences in within-group statistical comparisons (24). However, in our previous report on the effects of this nutritional intervention on body composition indices in a larger sample (7), minimal changes in body fat were observed in the control group, while results of the current study only show a comparable effect to the intervention group of the previous study that also lost significantly larger amounts of fat. So we expect that the effect of regression to the mean will here be minimal, if any. The same applies to the effect of the mere fact that one is engaged in a diet study *per se*, which could also have some nonspecific effects. This would include effects that may be due to weight loss rather than due to the specific intervention used to achieve the weight loss. In addition, our intervention was not limited to diet only because 45-min exercise sessions were offered to participants once to twice a week. Although in general an increase in physical activity alone may only have a small impact on obesity (25), we cannot fully dissociate the effects of the dietary intervention from the possible additional effects of enhanced physical activity in our study.

Our study has also several strengths. The participants were heterogeneous, self-selected, free-living, and without any personal preference toward PBD before study entry. The prescribed dietary intervention was self-financed; participants were not paid to be compliant to the targeted diet and were, on average, in a relatively good cardiovascular condition. We measured no negative or unexpected health effects, which is consistent with other studies that even used an increased protein intake of conventional plant-based protein (soy tofu) or protein supplementation (soy isolate protein), and found no harmful effects on liver and kidney function, bone mineral markers, and early stage prostate cancer progression (26–28). We were able to secure high levels of adherence through systematic follow-ups with testimonials, with the involvement of spouses and family members to support participants’ lifestyle changes, and probably also with the self-financing program of intervention diet. The reason why not all participants continued to the second phase of the study was probably due to the fact that they achieved most of the set goals (reduction of body weight, improved subjective level of energy, revealed improvements in biochemistry) and due to the burden of continuing to participate in the study (traveling, taking time to get to the laboratory, psychological factors when being committed to the study).

## Conclusion

Our single-arm interventional study has shown that an unrefined, low-fat, high-fiber PBD supplemented with plant-based MR in heterogeneous, self-selected, free-living, nonresidential conditions, eaten *ad libitum*, provides significant and meaningful improvements of total and LDL-cholesterol, non-HDL-cholesterol and body composition while maintaining safety with regard to kidney function, lean mass preservation, and blood cell counts. We have also shown that it is possible to achieve a very high compliance with this demanding intervention. However, a viable support program to keep close contact with participants is needed, especially in the first 2–3 months. The examined dietary pattern may present one viable option for achieving and maintaining long-term health in western countries with fast-paced conditions of modern life. This study is thus a valuable forerunner to a larger randomized study that should be done to confirm the proposed benefits of this nutritional paradigm.

## Conflict of interests and funding

Ba.J. is receiving royalty compensation at Herbalife Nutrition. Other authors declare no potential conflict of interest. Herbalife Nutrition had no role in the design of the study; in the collection, analyses, or interpretation of data; in the writing of the manuscript, and in the decision to publish the results.

J.P. received research grants to support his research work from Slovenian Research Agency (grant No. P3-0323). Slovenian Research Agency had no role in the design of the study and collection, analysis, and interpretation of data and in writing the manuscript.
